# *In vivo* Evaluation of Non-viral NICD Plasmid-Loaded PLGA Nanoparticles in Developing Zebrafish to Improve Cardiac Functions

**DOI:** 10.3389/fphys.2022.819767

**Published:** 2022-02-23

**Authors:** Victoria L. Messerschmidt, Uday Chintapula, Fabrizio Bonetesta, Samantha Laboy-Segarra, Amir Naderi, Kytai T. Nguyen, Hung Cao, Edward Mager, Juhyun Lee

**Affiliations:** ^1^Department of Bioengineering, University of Texas at Arlington, Arlington, TX, United States; ^2^University of Texas Southwestern Medical Center, Dallas, TX, United States; ^3^Department of Biological Sciences, University of North Texas, Denton, TX, United States; ^4^Department of Electrical Engineering and Computer Science, University of California, Irvine, Irvine, CA, United States

**Keywords:** PLGA nanoparticles, toxicity, non-viral transfection, zebrafish, gene delivery, Notch signaling

## Abstract

In the era of the advanced nanomaterials, use of nanoparticles has been highlighted in biomedical research. However, the demonstration of DNA plasmid delivery with nanoparticles for *in vivo* gene delivery experiments must be carefully tested due to many possible issues, including toxicity. The purpose of the current study was to deliver a Notch Intracellular Domain (NICD)-encoded plasmid *via* poly(lactic-*co*-glycolic acid) (PLGA) nanoparticles and to investigate the toxic environmental side effects for an *in vivo* experiment. In addition, we demonstrated the target delivery to the endothelium, including the endocardial layer, which is challenging to manipulate gene expression for cardiac functions due to the beating heart and rapid blood pumping. For this study, we used a zebrafish animal model and exposed it to nanoparticles at varying concentrations to observe for specific malformations over time for toxic effects of PLGA nanoparticles as a delivery vehicle. Our nanoparticles caused significantly less malformations than the positive control, ZnO nanoparticles. Additionally, the NICD plasmid was successfully delivered by PLGA nanoparticles and significantly increased Notch signaling related genes. Furthermore, our image based deep-learning analysis approach evaluated that the antibody conjugated nanoparticles were successfully bound to the endocardium to overexpress Notch related genes and improve cardiac function such as ejection fraction, fractional shortening, and cardiac output. This research demonstrates that PLGA nanoparticle-mediated target delivery to upregulate Notch related genes which can be a potential therapeutic approach with minimum toxic effects.

## Introduction

Since the early 2000s, more than 130 nanotechnology-based drug or delivery systems have been in clinical or pre-clinical studies (Duan et al., 2013). There have been numerous successes with differing delivery methods such as oral, local, topical, and intravenous delivery (Anselmo and Mitragotri, 2016). In fact, each mode of delivery mentioned has at least one FDA approved nanoparticle drug or delivery system (Barenholz, 2012; Cortajarena et al., 2014; Hua, 2015; Min et al., 2015). With the boom in nanotechnology, there is a greater need for more rigorous and meaningful pre-clinical studies using animal models to demonstrate the drug delivery efficiency validation.

The zebrafish (*Danio rerio*) are gaining traction as high throughput animal model that has easy maintenance and a fast developmental stage compared to other vertebrate animal models (Isogai et al., 2001; Bakkers, 2011). Additionally, many genes in the cardiovascular system and development are highly conserved between humans and zebrafish (Isogai et al., 2001; High and Epstein, 2008; Peshkovsky et al., 2011; Lee et al., 2016). Although the cardiac anatomical structure is significantly different, having one atrium and one ventricle, zebrafish have a similar electrophysiological signal and similar heart rate to that of humans at 110–130 beats per minute (bpm) (Hull et al., 2019). Alternatively, other small animal models, such as mouse, rats, and rabbits, have higher heart rates (Milani-Nejad and Janssen, 2014). Furthermore, by taking advantage of fluorescent technique, transparent zebrafish during early development gains leverage to study pharmacology, developmental biology, and molecular biology. For example, Smith et al. demonstrated that mutations in genes that were not previously related to congenital heart defects, but were then evaluated in zebrafish, showed that all 10 of the previously unrelated genes affected the atrioventricular canal, endothelial cushion, and valve malformations (Smith et al., 2009; Bakkers, 2011). Additionally, there are many congenital diseases that can be modeled in zebrafish to better understand the basic concepts of the genetic mutation (Bakkers, 2011).

Zebrafish are also a great model to evaluate toxicity of drugs or other treatments (Dai et al., 2014; Haque and Ward, 2018; Kumari et al., 2020; Elfawy et al., 2021; Verma et al., 2021). In addition to the high fecundity and transparency, their small size allows for multi-well assays to be easily used. As an important approach to validate nanomedicine using zebrafish, delivery through nanomaterial-based systems, such as PLGA nanoparticles, has been shown to reduce the adverse effects as well as to control the release of therapeutics by prolonging the drug while circulating in cardiovascular system (Thorley et al., 2014; Blanco et al., 2015; Chang et al., 2015; Messerschmidt et al., 2021b). However, translation of nanomedicines into clinical use is still a long-term challenge. Therefore, zebrafish are the first suitable animal model to evaluate the therapeutic approach of drugs and/or for testing toxicity to understand the molecular events of drug efficacy including siRNA delivery to injured zebrafish heart (Wang et al., 2019).

Even though there are numerous advantages to using zebrafish as a toxicology model, there are also limitations. First, chemicals or treatments are often dissolved into the fish media environment to expose them (Hill et al., 2005; Hu et al., 2011; Teijeiro-Valiño et al., 2017; Verma et al., 2021). The drawbacks to this method are that the treatments are administered or uptaken by methods that are not consistent with that of mammalian animal models (Cassar et al., 2020; Verma et al., 2021). For example, treatments in the liquid environment might be uptaken *via* the gills, oral route, or diffusion through the chorion depending on development stage. The differing exposure routes cannot be completely controlled by environmental exposure (Cassar et al., 2020; Verma et al., 2021). Additionally, drugs that degrade in water could expose the developing embryos to the break-down products of the intended treatment, rather than the treatment itself (Verma et al., 2021). Additionally, treatments that are not water-soluble cannot be easily administered in this manner, adding an additional complication (Verma et al., 2021). However, zebrafish studies have furthered *in vivo* toxicity experiments helped determine possible dosages for mammals and higher-level animal studies.

Notch signaling is essential for proper development of the heart, among other organs. However, it is also present in cancers, which requires that Notch manipulation by highly targeted in order to reduce potential side effects (Monticone and Miele, 2021). Notch is a membrane-bound protein that releases the Notch Intercellular Domain (NICD) once it is activated. The selectivity of the Notch signal depends on the concentrations of glycosyltransferases, and can alternate between delta-like ligands (DLL) or jagged (Jag) ligands (D’Amato et al., 2016). Additionally, Notch regulates cell signals between the endocardium and myocardium for chamber development (Chau et al., 2006; D’Amato et al., 2016). Without the cross communication, the heart cannot develop correctly.

Many groups have altered the expression levels of Notch to observe the effects. One group has forced Notch expression by utilizing retroviruses in chicks. Once the NICD is overexpressed, there was a reduction of cardiac myocyte markers (High and Epstein, 2008). Because of this down regulation, there were secondary effects that downregulated the vascular-endothelial (VE)-cadherin, which cause morphological changes (High and Epstein, 2008). Others have used knockdown techniques in zebrafish to show that without Notch, there are cardiac deformities (Milan et al., 2006; Bakkers, 2011). There are in depth review articles about Notch signaling and cardiac development (Chau et al., 2006; High and Epstein, 2008; Nemir and Pedrazzini, 2008; Niessen and Karsan, 2008; Bakkers, 2011; de la Pompa and Epstein, 2012; D’Amato et al., 2016).

Poly(lactic-co-glycolic acid) (PLGA) has been widely used as a biomedical material due to the cells ability to tolerate it and its break down products, lactic and glycolic acid (Makadia and Siegel, 2011). PLGA is popular as a drug carrier for proteins (Golub et al., 2010; Allahyari and Mohit, 2016; Haji Mansor et al., 2018), hydrophobic drugs (Shi et al., 2015; Allahyari and Mohit, 2016; Khan et al., 2016; Malinovskaya et al., 2017; Madani et al., 2018; Wilkosz et al., 2018; Lu et al., 2019; Choi et al., 2020) and hydrophilic drugs (Dalpiaz et al., 2016). More recently, PLGA has been used to carry genetic information to cells (Gvili et al., 2007; Cun et al., 2011; Amreddy et al., 2017; Guan and Rosenecker, 2017; Kalvanagh et al., 2019; Messerschmidt et al., 2021a). The various payload types speak to the versatility of PLGA and its’ ability to protect and deliver the contents to the body. Additionally, PLGA can be functionalized to further target specific cell lines (Kocbek et al., 2007; Fan et al., 2014; Shi et al., 2015; Khang et al., 2020; Messerschmidt et al., 2021a).

Like other target therapeutic delivery systems, the delivery of a nano-vehicle to the organ level *in vivo* remains a challenging issue (Barenholz, 2012; Min et al., 2015; Anselmo and Mitragotri, 2016, 2019). In this study, we aimed to investigate the toxicity level of PLGA nanoparticles to developing zebrafish if there is any as well as targeted delivery of Notch intracellular domain (NICD) plasmid by conjugation of PLGA nanoparticles with anti-Tie2+Tie1 antibody in order to upregulate the endothelial Notch signaling pathway. Furthermore, we evaluated the various types of environmental and physiological effects of PLGA nanoparticles to developing zebrafish and quantify cardiac functions by adopting deep-learning methods.

Herein, we consider it is essential to profile the binding and uptake of nanoparticles into the cellular level. We exposed zebrafish from 4to 96 hpf to varying concentrations of nanoparticles. A commonly used positive control of ZnO nanoparticles was utilized to determine the statistical relevance of genetic malformations (Hu et al., 2011; Duan et al., 2013; Teijeiro-Valiño et al., 2017). After a nanoparticle injection, the cardiac mechanics were evaluated as well as the transcriptional expression of Notch signaling related genes. Our data suggests that the PLGA nanoparticles are safe for developing zebrafish, as well as validates efficacy of the delivery vehicle by successfully upregulating Notch pathway related genes in an *in vivo* environment.

## Materials and Methods

### Zebrafish Husbandry

The zebrafish used for this study was raised and maintained in our zebrafish core facility under required regulations under the University of Texas at Arlington and University of North Texas Institutional Animal Care and Use Committee (IACUC) protocols. Wild-type AB Zebrafish (*Danio rerio*) embryos were collected after mating. To ensure a clear image, a medium composed of 0.0025% phenylthiourea (PTU) was used to suppress pigmentation at 20 h post-fertilization (hpf) (Lee et al., 2013). Every 24 h, the E3 media (CaCl_2_.2H_2_O 0.33 mM, MgSO_4_ 0.33 mM, NaCl 5 mM, KCl 0.17 mM, in 1 L of Milli-Q water) was exchanged, and the unfertilized or dead embryos were removed.

### Nanoparticle Preparation

TetO-FUW-NICD was a gift from Rudolf Jaenisch (Addgene plasmid #61540) (Cassady et al., 2014). Poly(D, L-lactide-*co*-glycolic acid) nanoparticles (PLGA, 50:50, 55–65 kDa, Akina Inc., West Lafayette, IN, United States) were fabricated by a standard double emulsion method as previously described (Messerschmidt et al., 2021b). In brief, 250 μg of plasmid was diluted in 5% glucose solution to 200 μL which was then emulsified into 0.5 mL of 5% (w/v) PLGA solution in chloroform using a probe sonicator at 40W energy output for 15 s to form a primary water/oil emulsion. The primary emulsion was then emulsified into 3 mL of 4% (w/v) PVA solution by sonication and later dropped into 7.5 mL of 0.3% (w/v) PVA solution while stirring. The final mixture was then stirred for 3 h at room temperature, and particles were collected by centrifugation. Nanoparticles were then lyophilized until completely dry before use. For a control, empty nanoparticles were loaded with only 5% glucose, no plasmid, following the same protocol as above.

### Nanoparticle Dosage and Toxicity Study

To evaluate the environmental toxicity effect of PLGA nanoparticles, zebrafish embryos were harvested at 4–5 hpf. Then, empty PLGA nanoparticles at various concentrations (2, 10, 25, 50 mg/mL) were suspend in E3 media mixed with methylene blue. E3 media alone served as a control. 50 embryos were placed in each petri dish with 25 mL of nanoparticle suspension. At 24−, 48−, 72− and 96-h post treatment (hpf), we performed a blind study to observe the hatching rate, survival rate, and malformations of embryos at each developmental stage after injection of different concentrations (*n* = 50). To distinguish the dead embryos, methylene blue added to E3 media and stained dead fish with a solid blue color while maintaining an antifungal condition. Malformations were the following: chorion with debris, delayed development, no movement at 24 h post treatment, pericardial edema, yolk sac edema, bent trunk, and tail malformations. At each time point, images were taken of each embryo for analysis. For malformation analysis, four individuals (high school volunteers) were asked to rank the malformations on a scale from 0 to 2, where 0 is no malformation, 1 when the malformation is present, and 2 the malformation is severe. All traits were added together to show the overall malformation score. A fish was considered “hatched” if it had completely broken out of the chorion. The following equations were used for hatching rate and survival rate:


$$H\,a\,t\,c\,h\,i\,n\,g\,R\,a\,t\,e=\,\frac{N\,u\,m\,b\,e\,r\,o\,f\,H\,a\,t\,c\,h\,e\,d\,F\,i\,s\,h\,p\,e\,r\,G\,r\,o\,u\,p}{T\,o\,t\,a\,l\,N\,u\,m\,b\,e\,r\,o\,f\,F\,i\,s\,h\,i\,n\,G\,r\,o\,u\,p}\times 100\%$$

$$S\,u\,r\,v\,i\,v\,a\,l\,R\,a\,t\,e=\,\frac{S\,u\,r\,v\,i\,v\,i\,n\,g\,F\,i\,s\,h\,P\,e\,r\,G\,r\,o\,u\,p}{T\,o\,t\,a\,l\,N\,u\,m\,b\,e\,r\,o\,f\,F\,i\,s\,h\,i\,n\,G\,r\,o\,u\,p}\times 100\%$$

### Zebrafish Injection

Zebrafish larvae were harvested at 48 hpf and incubated in buffered 0.003% tricaine until larvae were sedated to ensure proper anesthesia. The larvae were then placed in a 2% agarose E3 media mold laterally to visualize the heart and common cardinal vein (CCV) (Cosentino et al., 2010; Smith et al., 2015). All nanoparticles were at a concentration of 25 μg/mL in saline (0.9% NaCl) and injected using a total volume of 5 nL. Doxycycline was added to E3 media to induce translation of the TetO-FUW-NICD plasmid at a final concentration of 2 μg/mL.

### *In vivo* Bio-Distribution

Through the CCV of 48 hpf zebrafish larvae, we have injected coumarin 6-loaded Anti-Tie2+Tie1 conjugated PLGA nanoparticles suspended in saline. After 2 h, 10× images were taken to observe the location of the nanoparticles if nanoparticles were successfully targeted in vascular system. Videos at 10× magnification (IX-73, Olympus, Tokyo, Japan) were also taken, shown as representative stills, to depict nanoparticles in the circulatory system (Supplementary Video 1).

### Reverse Transcriptase Quantitative PCR

Zebrafish larvae were harvested at 48 hpf and injected into the CCV with the following groups: saline (0.9% NaCl), or saline with NICD-loaded Anti-Tie2+Tie1 conjugated PLGA nanoparticles. The stability of our plasmid loaded PLGA nanoparticles was assessed prior to injections by measuring the size using Dynamic Light Scattering (DLS) technique in both saline and complete cell growth media with 10% serum. The nanoparticles were injected with 25 μg/mL, based on the tested toxicity performed. After 12 h post injection (hpi) (at 2.5 days post fertilization = dpf zebrafish), 24 hpi (at 3 dpf zebrafish) and 48 hpi (at 4 dpf zebrafish), zebrafish were sacrificed with an overdose of tricaine. The whole zebrafish was then homogenized by a homogenizer (Bertin Instruments, Montigny-le-Bretonneux, France), and RNA samples were isolated using Bio-Rad’s Aurum Total RNA Mini Kits. cDNA was synthesized using Bio-Rad’s iScript cDNA Synthesis kits. PCR was conducted in triplicate with the primers described in Lee et al. (2016). In addition, to understand gene expression level changes in the hearts, we have bred and setup the same experiment as above. Then, the embryonic hearts were isolated under a dissecting microscope, and RT-PCR was run as previously described in Yang and Xu (2012).

**Table:** 

Target	Forward primer	Reverse primer
*zNICD*	GCAGGATCCACCATGGGTTGTG GGGTGCTGCTGTCCCGCAAG	CTTGAATTCTTACTTAAATGCC TCTGGAATGTGGGTG
*zNotch1b*	CAGAGAGTGGAGGCACA GTGCAATCC	GCCGTCCCATTCACACTC TGCATT
*zDLL4*	CAAAGTGGGAAGCAGACA GAGCTAAGG	CGGTCATCCCTGGGTG TGCATT
*zHes1*	GAGAGGCTGCCAAGGTTTTT	GTAATACGACTCACTATAGGGT CAAATAAACTTCCCCAAAGGA
*zHey1*	AAACGTCGCAGAGGGATCAT	CCTGTTTCTCAAAGGCGCTG

### Cardiac Function Analysis From Bright Field Image by Deep-Learning Network

Zebrafish larvae were harvested at 48 hpf and injected into the CVV with the following groups: saline (0.9% NaCl), blank PLGA nanoparticles in saline, NICD-loaded PLGA nanoparticles in saline, and NICD-loaded Anti-Tie2+Tie1-conjugated PLGA nanoparticles in saline. After 24 hpi (3 dpf), 48 hpi (4 dpf), 72 hpi (5 dpf), zebrafish were anesthetized with 0.003% tricaine and placed in the 2% agarose E3 media mold laterally. The heart was visualized and viewed on a microscope at 4× magnification. Videos were taken in a bright field of the heart for analysis of ejection fraction (EF), fractional shortening (FS), end-systolic (ES) and end-diastolic (ED) volume by the deep-learning framework Zebrafish Automatic Cardiovascular Assessment Framework (ZACAF), which was recently developed and validated (Naderi et al., 2021).

For segmentation, briefly, ZACAF utilizes a deep learning model with a U-net architecture, which was designed for sematic segmentation in biomedical imaging. The model was trained to segment the ventricle in bright-field frames of a microscopic video from a zebrafish. First, each frame of an input video will be preprocessed with a sharpening filter to improve the edges and with contrast limited adaptive histogram equalization (CLAHE), which is used for improving the visibility level in each frame. Second, the trained U-net segments the ventricle in all frames. The output of the U-net is a binary black and white image where the pixels detected as ventricle are black and rest of the pixels are white. Counting the number of the black pixels will result in the area of the ventricle. The maximum and minimum measured areas of the ventricle in different frames show the ES and ED stages, respectively. Having the measurement of ES and ED frames, ejection fraction (EF), fractional shortening (FS), and cardiac output (CO) were calculated. Heart rate from each fish was manually counted from videos. The predicted ventricle is assumed to be an ellipsoid. For quantification of EF, the following formula (Yalcin et al., 2017) is used: $E\,F\%=\frac{\left(E\,D\,A-E\,S\,A\right)}{E\,D\,A}\times 100\%$ (1); where the EDA and ESA are the area of the ventricle in ED and ES, respectively. Since the frames are 2D images, we have to estimate the ventricle volume to its area. For calculating FS, measurements of the short axis in ES and ED frames are taken (Naderi et al., 2021).

### Statistical Methods

All statistics were evaluated in the statistical program R unless otherwise noted. For the malformations, a one-way ANOVA was conducted followed by a Tukey test at each time point (24, 48, 72, or 96 h). The cardiac mechanical performance measures were evaluated for normality, equal variance, and subsequently evaluated between the two groups using either parametric *T*-Test or non-parametric Wilcoxon Ranked Sum Test. The survival test was conducted using GraphPad Prism 9.2.0 (San Diego, CA, United States). Bonferroni Correction was used to correct for multiple comparisons. RT-PCR results were evaluated using a one-way ANOVA per gene followed by a Tukey test. All *p*-values lower than 0.05 were considered to be significant.

## Results

### Nanoparticle Dosage and Toxicity Study

**FIGURE 1:**
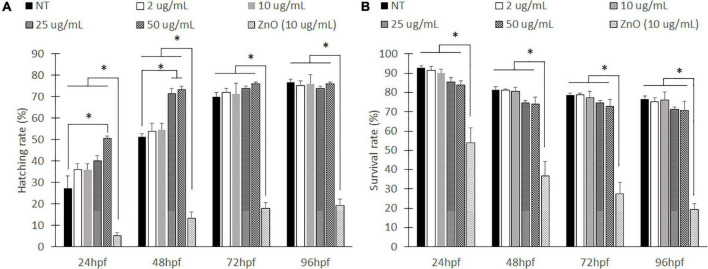
Hatching rates and survival rate for embryonic zebrafish toxicity study. Hatched to unhatched ratio **(A)** and survival rate **(B)** of non-treated fish to various concentrations of empty PLGA nanoparticle suspension. *indicates a significant difference (*p* < 0.01). *n* = 50 fish per group.

To test the environmental effects of PLGA nanoparticles on developing zebrafish, we first exposed wild-type zebrafish embryos to various concentrations of PLGA nanoparticles suspended in E3 media from 2 to 50 μg/mL whereas ZnO nanoparticles at 10 μg/mL served as a positive control (Hu et al., 2011; Teijeiro-Valiño et al., 2017). ZnO nanoparticles were chosen due to their popularity in inducing developmental irregularities (Hu et al., 2011; Choi et al., 2016; Teijeiro-Valiño et al., 2017). At 10 μg/mL, there are physical abnormalities that can be seen with a stereoscope, allowing for easily identifiable malformations. After 24 h, there was a significant difference in hatching rate from the chorion between all nanoparticle exposed groups and the ZnO nanoparticle exposed group (Figure 1A). A low hatching rate of ZnO nanoparticle exposed group remained until 96 hpf. On the other hand, at 24 h, all exposed PLGA nanoparticle groups caused early hatching compared to the only E3 treated group, which is no treatment (NT) of any nanoparticles. Specifically, with the exposure of 25 and 50 μg/mL concentrations of PLGA nanoparticles, zebrafish embryos were hatched significantly earlier than NT at 48 hpf. However, as treatment time prolonged, the hatching rate of all concentrations of PLGA nanoparticles became insignificant to NT.

Zebrafish viability was also evaluated every 24 h (Figure 1B). The ZnO nanoparticles caused a significant decrease in survival rates compared to all other groups. The ZnO particles had less than 20% of the survival rate at 96 hpf. However, zebrafish exposed to PLGA nanoparticles did not show significant differences of viability compared to NT. Although the higher nanoparticle concentrations, 25 and 50 μg/mL, have slightly lower compared to NT at an earlier time (24 and 48 hpf), they were not significant, and survival rate became similar to NT at 96 hpf. Compared to the no treatment group, the PLGA nanoparticles did not seem to cause a significant effect.

Various malformations were observed in the zebrafish embryos over time including chorion debris, delayed development, pericardial edema, yolk sac edema, bent trunk, and tail malformations. Within the first 24 h, there was no significant difference of PLGA nanoparticle treated groups and NT. However, the positive control, ZnO nanoparticles, caused significantly more chorion debris than the other groups (Supplementary Figure 1A). The ZnO nanoparticles also caused significantly more developmental delay at all developmental stages, including death, compared to the other groups such as PLGA nanoparticles (Supplementary Figure 1B). There was also less significant pericardial edema and yolk sac edema in the PLGA nanoparticle treated groups compared to control group, ZnO (Supplementary Figures 2A,B). As well as testing other developmental defects, the PLGA nanoparticle treatment did not cause the malformation of bent trunk and tail formation while the ZnO nanoparticles affect significant developmental defects (Supplementary Figure 3). Therefore, the PLGA nanoparticles is a safe nanomaterial in the aquatic developmental environment.

### Bio-Distribution of PLGA Nanoparticles

**FIGURE 2:**
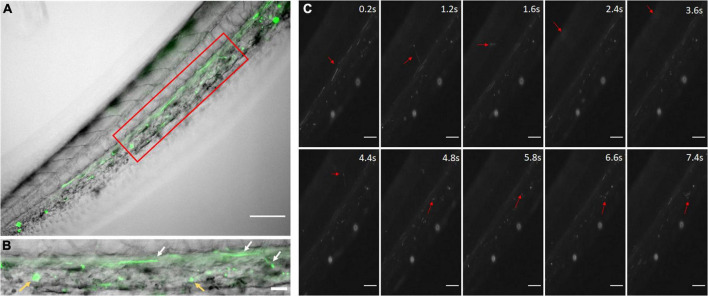
Coumarin-6 Loaded Nanoparticles after Injection. **(A)** Coumarin-6 nanoparticles traveling in the caudal artery of the zebrafish tail. Red box is blown up and shown as **(B)**. White arrows show nanoparticles rolling along vessel walls. Yellow arrows indicate clusters of endocytosed nanoparticles. **(C)** Representative stills showing nanoparticles traveling from the caudal artery (0.2–1.2 s), into the capillaries of the tail tissue (1.6–4.8 s) and entering the posterior caudal vein (6.6–7.4 s). All scale bars represent 100 μm.

Before *in vivo* experiment, we have characterized the properties of PLGA nanoparticles and tested *in vitro* with HUVECs (Messerschmidt et al., 2021b). In addition, there were no significant changes in size of NICD plasmid loaded PLGA nanoparticles over time in both saline and cell growth media with serum proteins (Supplementary Figure 4). This shows good stability of our NICD plasmid loaded PLGA NPs in saline (to preparing NP suspensions for IV administration) and also in the presence of serum proteins which mimics the *in vivo* environment encountered by NPs after administration *via* IV up to 96 h. To demonstrate the circulation of nanoparticles in the zebrafish vascular system, coumarin-6 loaded nanoparticles were injected and imaged with a fluorescent microscope. The nanoparticles were easily observed as fast-moving particles in the blood stream (Figure 2A). By 2 h, small clusters of nanoparticles had been uptaken by the zebrafish (Figure 2B). Rolling nanoparticles could be seen as long streaks on the walls of vessels (Supplementary Video 1). Uptaken nanoparticles were depicted as stationary fluorescent clusters (Figure 2B, yellow arrows). The fluorescent nanoparticles could be seen traveling down the tail *via* the dorsal aorta into the caudal artery, into the caudal vein, and up the posterior cardinal vein. Nanoparticles were also able to enter the capillaries and travel into the deeper tissues (Figure 2C). As the blood velocity slows down, nanoparticles also travel at a slower speed (1.6–4.8 s). Once nanoparticles neared the posterior cardinal vein, the nanoparticles’ speed increased.

**FIGURE 3:**
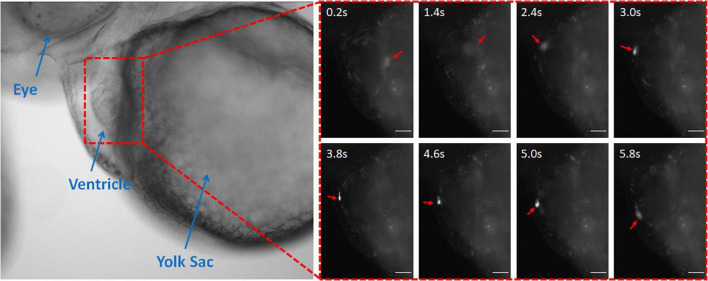
Coumarin-6 Nanoparticles Rolling on Endocardium. Representative stills showing nanoparticles flowing through the cardiac chambers. Arrows indicate injected nanoparticle. All scale bars represent 100 μm.

The coumarin-6 nanoparticles were also visible in the zebrafish heart. The nanoparticles could be seen rolling along the endocardium and the heartbeat (Figure 3 and Supplementary Video 2). The observed nanoparticles were bound more tightly to the endocardium, slowing their speed (3.0–5.0 s). However, the unbound nanoparticles flew through the rest of the zebrafish as fast as in vascular system due to rapid blood velocity and motion of the cardiac wall. Therefore, for effective targeting, the conjugation technique with antibodies would be more effective (Messerschmidt et al., 2021b).

### Upregulation of Notch by Injecting Notch Intracellular Domain Plasmid Loaded PLGA Nanoparticles

**FIGURE 4:**
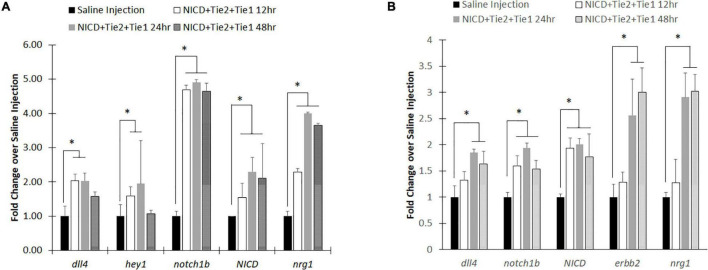
RT-PCR analysis after injecting intravenous NICD loaded nanoparticles. **(A)** Notch signaling components were all upregulated after injection of NICD loaded nanoparticles until 24 h post injection. However, Notch ligand (*dll4*) and receptor (*notch1b*) were returned to control group (saline injection) level while downstream components (*nrg1* and *hey1*) remain high with *NICD* expression level. **(B)** Isolated zebrafish heart after injection showed higher expression levels of Notch components to increase cardiac function. Interestingly, endocardial *nrg1*-myocardial *erbb2* signaling components consistently increased up to 48 h post injection. * indicates a significant difference (*p* < 0.01). *n* = 5.

For effective targeting, we have conjugated the nanoparticles with Tie2+Tie1 antibodies for endothelium targeting. Additionally, we loaded NICD plasmid to our PLGA nanoparticles to upregulate Notch signaling for the zebrafish *in vivo* experiment. From our previous work, we demonstrated that Tie2+Tie1 antibody conjugated PLGA nanoparticles successfully upregulated Notch related genes within the *in vitro* flow channel with HUVECs (Messerschmidt et al., 2021b). We also optimized NICD loaded PLGA nanoparticle concentration, 5 nL of 25 μg/mL, and injected at 48 hpf of zebrafish through the CCV. NICD-loaded Anti-Tie2+Tie1-conjugated nanoparticles were compared to saline injection group. Through whole-body qRT-PCR, the NICD loaded nanoparticles had significantly higher mRNA expression of Notch pathway related genes at each time point compared to the saline injection (Figure 4A). Additionally, mRNA expression peaked at 24 h post injection for all genes observed. After 48 h, *dll4* (Notch ligand) and *Notch1* (Notch receptor) returned to insignificant levels while both downstream Notch genes, *hey1* and *nrg1*, continued to show significantly higher expression after 48 h post injection compared to saline. Lastly, *NICD* was significantly higher at 48 h with a high standard deviation, implying that the nanoparticles were still releasing NICD plasmids by the slow degradation depending on PLGA nanoparticles’ molecular weight.

To evaluate whether PLGA nanoparticles can be delivery vehicles to the heart, we redo the experiment with new group of zebrafish. This time, we dissected zebrafish hearts from each of the developmental stages after the nanoparticle injection to evaluate the expression levels of Notch related genes. Previous injections of nanoparticles without Tie2+Tie1 antibodies conjugation were not taken effectively into the endocardium due to dynamic motion and rapid blood pumping. However, with conjugation of antibodies, nanoparticles were uptaken into the endocardium and successfully upregulated Notch related transcripts (Figure 4B). Similar to whole-body analysis, *dll4, notch1b*, and *NICD* expression levels were the highest at 24 h post injection. Interestingly, Notch downstream signaling, endocardium *nrg1*-myocardium *erbb2* shows the further overexpression at 48 hpf, implicating increasing cardiac mechanics by injecting our nanoparticles maintained.

### Cardiac Mechanical Performance

**FIGURE 5:**
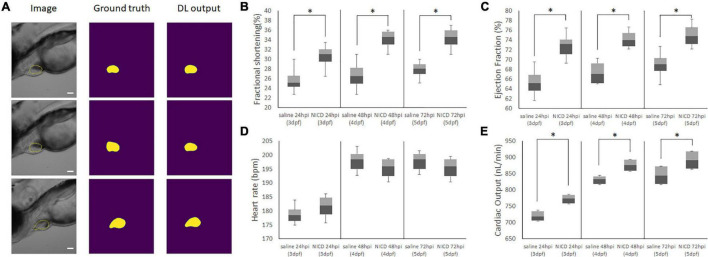
Representative Images of Model Output for analyzing cardiac functions. **(A)** Representative images of the image input (column 1), hand segmented cardiac volume (column 2), and the model’s predicted cardiac volume after training the ZACAF network (column 3). **(B,C)** FS and EF comparisons demonstrated the zebrafish heart contracts stronger after injecting NICD incorporated PLGA nanoparticles. **(D)** Injection of NICD loaded PLGA nanoparticles didn’t affect heart rate. **(E)** Cardiac output of both control group and NICD loaded nanoparticles gradually increased as zebrafish heart matures. However, nanoparticles injected zebrafish has higher number of cardiac output. * indicates a significant difference (*p* < 0.01). *n* = 250 zebrafish images per group.

To understand the impact of our NICD loaded nanoparticles to cardiac mechanics, we introduced a deep learning algorithm to evaluate the cardiac performance by analyzing bright images from an inverted microscope (Yalcin et al., 2017; Figure 5A). As training data, we have hand-segmented zebrafish ventricle as a ground truth mask. After training our deep-learning network, the dice loss coefficient plateaued at 0.18. Predictions of the zebrafish heart shape after training was provided more accurate segmentation by reducing inconsistency of inter-rater reliability (Supplementary Figure 4A). Additionally, the Intersection of Union (IoU) score of deep-learning network (ZACAF) reached 0.80 (Supplementary Figure 4B). The model can be qualitatively compared to show that the model prediction is similar to the hand segmented images (Naderi et al., 2021).

From the model, we analyzed the data with box and whiskers to visualize all the data distribution from the deep-learning algorithm. By measuring the end systolic area (ESA) and end diastolic area (EDA) counting voxels, we were able to calculate ejection fraction (EF), fractional shortening (FS), and cardiac output (CO) by counting heart rate (Figures 5B–E). We found the NICD plasmid incorporated PLGA with Tie2+Tie1 antibodies injected fish had a larger chamber volume size than the saline injected fish, and its fractional shortening (FS), ejection fraction (EF), cardiac output (CO) were also increased. Although analysis of cardiac mechanics has an increasing trend after injection of nanoparticles, there was no significant change in heart rate. This data shows delivery of NICD plasmids successfully upregulated downstream of Notch and improved the cardiac functions by increasing contractile forces.

## Discussion

While PLGA is a commonly used biopolymer in medical research as a drug delivery or therapeutic material, assessing exposure effects in a dose-dependent manner in aquatic conditions remains elusive. In this work, we demonstrated PLGA nanoparticles are safe delivery vehicles for zebrafish. In addition, we demonstrated PLGA nanoparticles can be an effective plasmid delivery vehicle to upregulate gene expression using the developing zebrafish model. From our previous *in vitro* experiment (Messerschmidt et al., 2021b), we have optimized the concentration of PLGA nanoparticles to the HUVEC cells and efficacy of delivering NICD to upregulate the Notch pathway. Using intravenous injection of PLGA nanoparticles into zebrafish, we visualized rapid uptake and therapeutic effect on Notch to improve cardiac functions by analysis with deep-learning methods. Previously, PLGA nanoparticles have been used as a safe material for therapeutic applications (Fenaroli et al., 2014; Vibe et al., 2016). However, environmental effects remained questionable to test if the exposure of PLGA polymer for long-term, 3 days, is still harmless for developing conditions although it is biodegradable material. Although there was only a significant difference of chorion hatching rate between NT and 50 μg/mL of PLGA nanoparticle exposed groups, the trend of hatching rate increased gradually causing minimal effects (Figure 1A). Early dechorionated zebrafish exposed to a high concentration of PLGA nanoparticles (50 μg/mL) shows insignificant difference in pericardial edema and yolk sac edema, showing a severity level of 0.3 out of 2, indicating that we could ignore this insignificant level. However, an earlier study showed premature dechorionated embryos could be vulnerable to environmental toxicity, such as pesticides or drugs, resulting in teratological and behavioral effects (Bodewein et al., 2016; Dach et al., 2019). Despite PLGA nanoparticles are known to be a biodegradable and a safe material that is harmless to developing zebrafish body, the secondary effect of early dechorionization could cause zebrafish developmental malformation if the concentration is more than 50 μg/mL.

As a delivery vehicle to upregulate specific genes, we loaded NICD plasmid into the PLGA nanoparticle and injected through zebrafish CCV with microinjections to demonstrate bio-distribution and efficacy of upregulation of Notch pathway molecules. As early as 12 h post injection, there was a significant increase in *hey1*, *notch1b*, *NICD* and *nrg1* genes. At 24 h, all genes were significantly upregulated. The intense upregulation of *notch1b* and its related genes could be due to the burst release of plasmid shown in our previous work (Messerschmidt et al., 2021b). The similarities between this work and previous ones indicate that our model drug and release profiles from Messerschmidt et al. (2021b) are effective in predicting the outcomes *in vivo* (Messerschmidt et al., 2021b). Additionally, the western blot and RT-PCR results from our previous *in vitro* data are mirrored in the upregulation of the same genes in the zebrafish model (Messerschmidt et al., 2021b). However, after 24 hpf, *dll4* and *notch1b* returned to insignificant level similar to saline injection (Figure 4A). There are two possible reasons. First, the amount of time for the feedback loop to return to the normal level by homeostasis occurs to lower expression levels in healthy zebrafish (Lin and Amir, 2018). At the cellular level, receptor activity that elicits up-regulation of gene expression by negative feedback mechanisms is commonly found (Lin and Amir, 2018). Second, while nanoparticles circulate in the blood stream, renal filtration or opsonization by mononuclear phagocyte system (MPS) might clear the injected nanoparticles (Alexis et al., 2008; Longmire et al., 2008). In fact, the size of the nanoparticles matter during circulation. Therapeutic nanoparticles are in 20–200 nm size to avoid the filtration system by kidney in human (Ernsting et al., 2013; Wu et al., 2018). However, the small size of zebrafish embryos may be more sensitive to nanoparticles with the size of ∼200 nm (Messerschmidt et al., 2021b). Interestingly, once nanoparticles were uptaken and released NICD, downstream genes remained expressed after 48 h post injection (96 hpf). Furthermore, downstream genes of the Notch pathway were successfully expressed in zebrafish, as well as exhibited the phenotype for functional improvement (Lee et al., 2018). Our work demonstrated that *erbb2* in myocardium was over-expressed by upregulated endocardial Notch and increased cardiac functions (Figures 4B, 5). Although increased hemodynamic force due to higher viscosity by injecting nanoparticles could be a factor for increased Notch signaling since Notch is mechanosensitive, Lee et al. showed that increased particle number would not be significant enough to upregulate Notch (Lee et al., 2018). Therefore, upregulation of Notch signaling in our study is by NICD gene delivery with PLGA nanoparticles.

## Conclusion

In conclusion, we have shown that PLGA nanoparticles less than 50 μg/mL do not have significant effect on the physical conditions of zebrafish when zebrafish were exposed to PLGA nanoparticles environmentally. Although the high concentration nanoparticles induced a rapid hatching, this does not affect the survival rates of the zebrafish during developmental stage. Additionally, after successful intravenous injections of the targeting nanoparticles with Tie2+Tie1 antibodies, we were able to demonstrate that the treatment significantly upregulated Notch signaling pathways as well as improving the cardiac functions. The nanoparticles were successfully uptaken after rolling on the endothelial lining and endocardium by interacting with the Tie1 or Tie2 proteins expressed on those cells. Therefore, the PLGA nanoparticles are a useful therapeutic plasmid delivery vehicle for zebrafish research, as well as safe for future cardiovascular studies.

## Data Availability Statement

The original contributions presented in the study are included in the article/Supplementary Material, further inquiries can be directed to the corresponding author.

## Ethics Statement

The animal study was reviewed and approved by University of Texas at Arlington and University of North Texas.

## Author Contributions

VM and FB: *in vivo* experiment and analysis. VM and UC: nanoparticle fabrication, characterization, and optimization. VM and AN: deep-learning analysis for cardiac function. VM, UC, and SL-S: nanoparticle stability. HC and JL: deep-learning supervision. KN and JL: nanoparticle preparation supervision. EM and JL: supervision of in vivo analysis of zebrafish after injection of nanoparticles.

## Conflict of Interest

The authors declare that the research was conducted in the absence of any commercial or financial relationships that could be construed as a potential conflict of interest.

## Publisher’s Note

All claims expressed in this article are solely those of the authors and do not necessarily represent those of their affiliated organizations, or those of the publisher, the editors and the reviewers. Any product that may be evaluated in this article, or claim that may be made by its manufacturer, is not guaranteed or endorsed by the publisher.
