# Supplementary material for: In vivo Evaluation of Non-viral NICD Plasmid-Loaded PLGA Nanoparticles in Developing Zebrafish to Improve Cardiac Functions
Source: Front Physiol. 2022 Feb 23;13:819767. doi: 10.3389/fphys.2022.819767 (PMC8906778; doi:10.3389/fphys.2022.819767)
Supplement: Supplementary file 3 [file Data_Sheet_1.PDF]

Supplementary document

In vivo evaluation of non-viral NICD plasmid-loaded PLGA nanoparticles in developing zebrafish to improve cardiac functions

Victoria L. Messerschmidt[1,2], Uday Chintapula[1,2], Fabrizio Bonetesta[3], Samantha Laboy-Segarra[1,2], Amir Naderi[4], Kytai T. Nguyen[1,2] , Hung Cao[4], Edward Mager[3], Juhyun Lee[1,2]

[1]Department of Bioengineering, University of Texas at Arlington, Arlington TX 76010 USA

[2]University of Texas Southwestern Medical Center, Dallas TX 75390 USA

[3]Department of Biological Sciences, University of North Texas, Denton TX 76203

[4]Department of Electrical Engineering and Computer Science, University of California Irvine, Irvine CA 92697 USA

Corresponding Author:

Juhyun Lee, Ph.D.

Joint Department of Bioengineering

UT Arlington / UT Southwestern

Arlington, TX 75022

Email: juhyun.lee@uta.edu

Telephone: 817-272-6534

Fax: 817-272-2251

Figure S1: Chorion debris formation and developmental delay on zebrafish embryos.

Figure S2: Yolk sac and pericardial edema after nanoparticle exposure.

Figure S3: Development of bent trunk and tail malformations after nanoparticle exposure.

Figure S4: NICD plasmid loaded PLGA NPs stability in saline and complete cell growth media.

Video S1: Circulating nanoparticles in vascular system.

Video S2: Circulating nanoparticles inside of zebrafish heart.

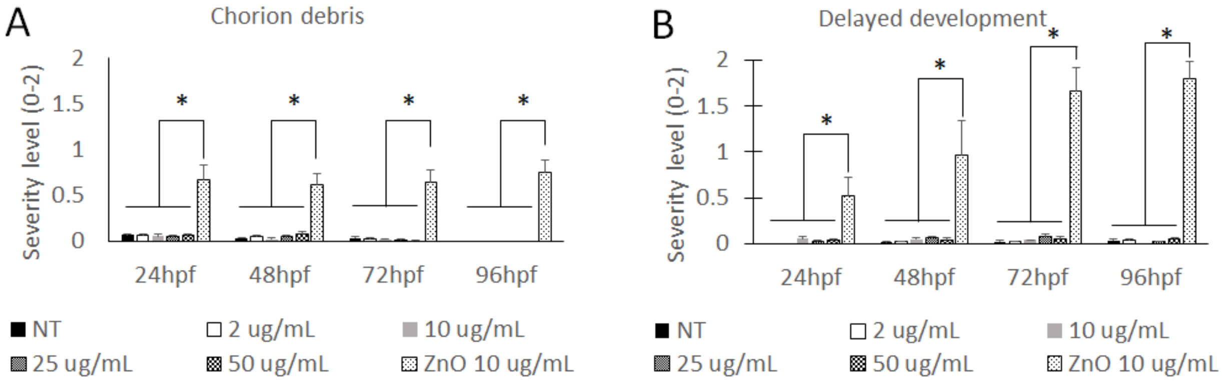

Figure S1: Chorion debris formation and developmental delay on zebrafish embryos. (A) Chorion debris was rated 0, 1, or 2 based on the severity of the debris every 24 hours. (B) Severity level of developmental delay was also scored from 0 to 2 by blind testing. * indicates a significant difference from ZnO within time point ($p<0.01$). n = 50 fish per group.

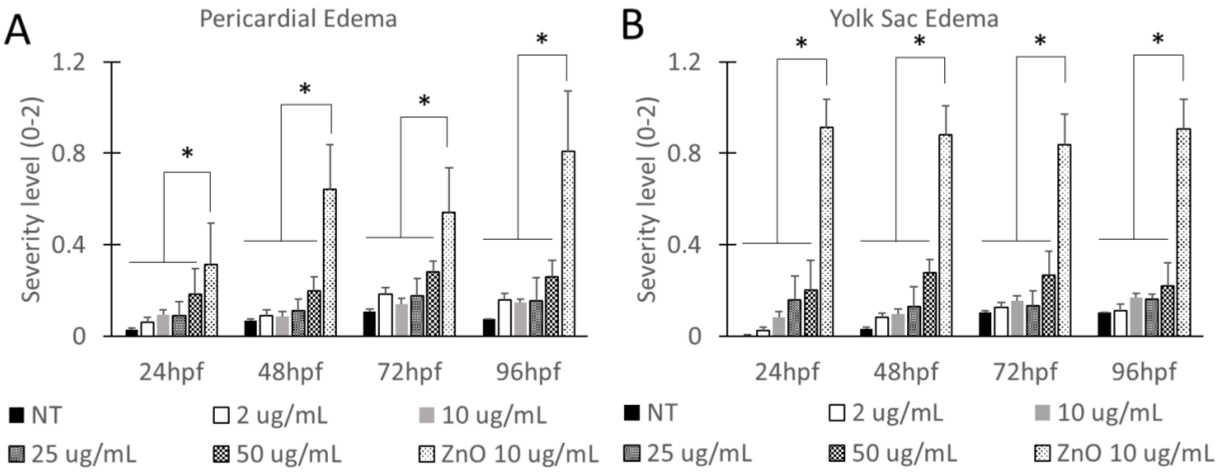

Figure S2. Yolk sac and pericardial edema after nanoparticle exposure. (A) Severity level of pericardial edema is not significant when zebrafish is developing. Although, at 24 hpf, the higher concentrations (25 μg/mL to 50 μg/mL) of PLGA nanoparticle treated group doesn't have significant difference in pericardial edema comparted to ZnO treated group. However, the severity level is low at this developmental stage. (B) ZnO nanoparticle treated group caused significantly higher yolk sac edema from 24 hpf. * indicates a significant difference from ZnO within time point ($p<0.01$). n = 50 fish per group**.**

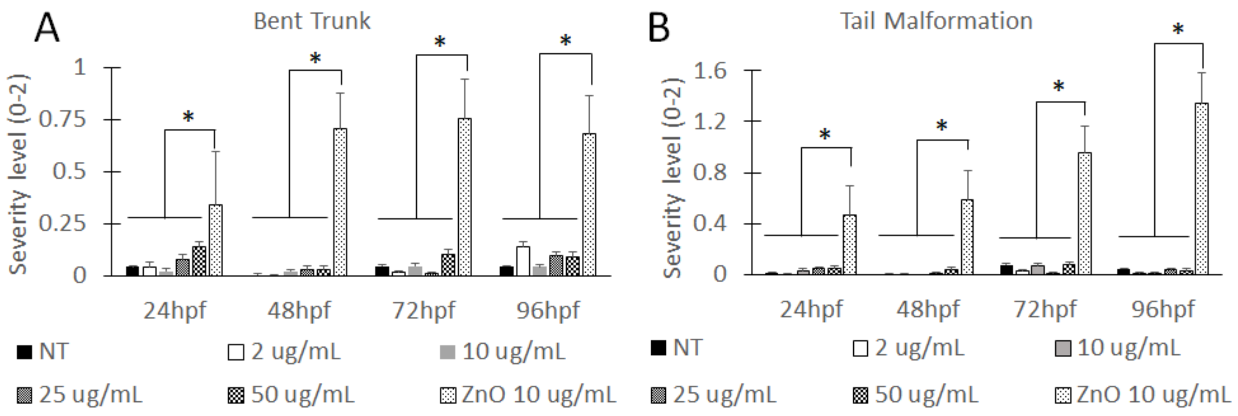

Figure S3. Development of bent trunk and tail malformations after nanoparticle exposure. (A) Severity level of bent trunk is significantly higher in ZnO nanoparticles while PLGA nanoparticle treated group has no significant difference compared to NT. (B) Tail formation also has no significant difference between NPs and NT, while ZnO nanoparticle caused significant defect in tail formation. * indicates a significant difference from ZnO within time point ($p<0.01$). n = 50 fish per group.

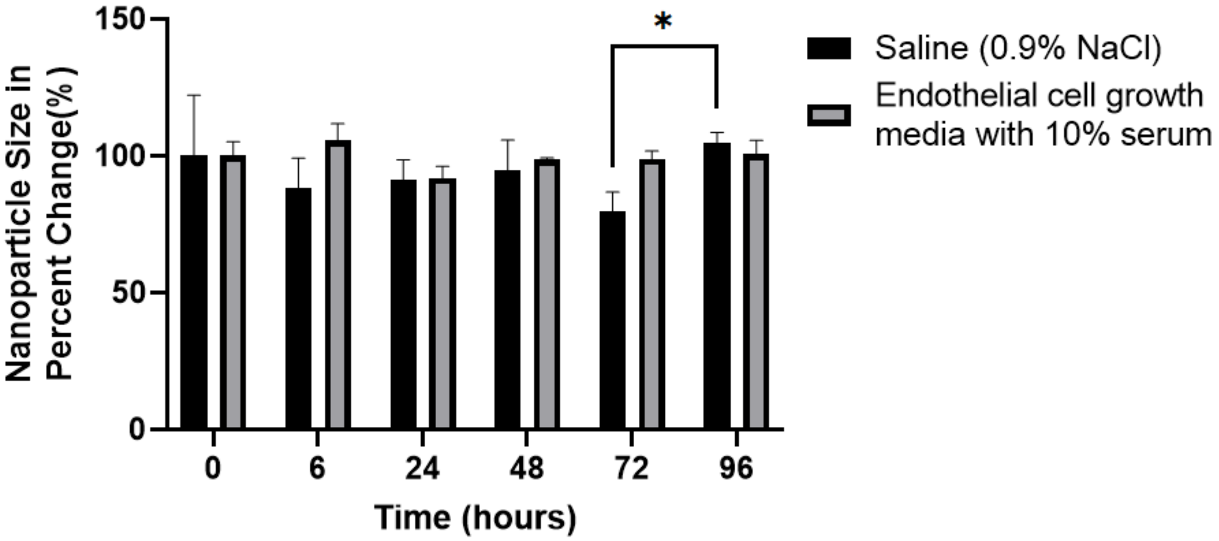

Figure S4. NICD plasmid loaded PLGA NPs stability in saline and complete cell growth media. NICD plasmid loaded PLGA nanoparticle stability in saline (0.9% NaCl) or in cell growth media with 10% serum over time shows no significant changes except some variation in size seen after 72hrs in saline. Error bars denote standard error. * Indicates a significant difference of sizes between the highlighted timepoints in saline group ($p < 0.05$, n=3). Statistical Analysis was done using Sidak's multiple comparisons test.
